# SRC Kinase-Mediated Tyrosine Phosphorylation of TUBB3 Regulates Its Stability and Mitotic Spindle Dynamics in Prostate Cancer Cells

**DOI:** 10.3390/pharmaceutics14050932

**Published:** 2022-04-25

**Authors:** Alan Alfano, Jin Xu, Xi Yang, Dhanraj Deshmukh, Yun Qiu

**Affiliations:** 1 Department of Pharmacology, University of Maryland School of Medicine, Baltimore, MD 21201, USA; aalfa001@umaryland.edu (A.A.); jinxu@umaryland.edu (J.X.); xyang@som.umaryland.edu (X.Y.); dhanrajdeshmukh@umaryland.edu (D.D.); 2 Veterans Affairs Medical Center, Baltimore, MD 21201, USA

**Keywords:** tubulin, TUBB3, SRC kinase, tyrosine phosphorylation, microtubule, prostate cancer, drug resistance

## Abstract

Tubulin is an integral part of the cytoskeleton and plays a pivotal role in cellular signaling, maintenance, and division. β-tubulin is also the molecular target for taxane compounds such as docetaxel (DTX) and cabazitaxel (CTX), both first-line treatments for several solid cancers. Increased expression of Class III β-tubulin (TUBB3), a primarily neural isoform of β-tubulin, correlates with taxane resistance and poor prognosis. Although tyrosine kinase c-Src has been implicated to phosphorylate β-tubulins during both hematopoietic and neural differentiation, the mechanisms by which Src modulates tubulins functions are still poorly understood. Here, we report, for the first time, that TUBB3 is phosphorylated at Tyrosine 340 (Y340) by c-SRC in prostate cancer cells. We also showed that Y340 phosphorylation regulates TUBB3 protein stability and subcellular localization. Furthermore, we demonstrated that inhibition of SRC kinase activity compromises spindle stability in mitotic cells, at least partly due to the lack of TUBB3 Y340 phosphorylation. Given the importance of TUBB3 as a clinical biomarker of poor prognosis and drug resistance, characterization of TUBB3 posttranslational regulation could potentially serve as new biomarkers for disease recurrence and/or treatment failure.

## 1. Introduction

Prostate cancer (PCa) is the most common male malignancy in the United States and the second most lethal cancer in men [1,2,3]. For patients with diagnoses of advanced PCa, the standard treatment is androgen deprivation therapy (ADT). Androgen deprivation is achieved either by radical prostatectomy or by chemical castration [2,3]. Although ADT is initially successful at reducing tumor burden and circulating PSA levels, many PCa patients experience a relapse into an incurable, androgen-insensitive disease known as Castration-Resistant Prostate Cancer (CRPC) (1–3). Taxane compounds (i.e., Docetaxel and Cabazitaxel), Abiraterone, and sipuleucel-T are the current treatments FDA approved for CRPC, with Docetaxel (DTX) + Steroid (such as Prednisone) being the first-line treatment [4]. Though current treatment options offer some survival benefits (3–6 months on average), drug resistance is a common problem in advanced CRPC patients [2,3,4]. Progression to CRPC and eventual failure of CRPC treatments remain the major cause of prostate cancer lethality. Thus, they represent critical barriers to the effective management of advanced PCa. A deeper understanding of sub-cellular processes involved in castration resistance and eventual drug resistance is critical for the improvement of current PCa treatment regimens. Additionally, characterizing novel signaling events in drug-resistant CRPC could lead to improved biomarkers for diagnosis and/or new points of intervention for future therapies. 

The SFK (Src-family kinases) family consists of 9 non-receptor tyrosine kinases. They are also the molecular targets of several chemotherapeutic agents in clinical use, such as dasatinib [5]. C-Src, the prototypical SFK, is one of the oldest known and most widely studied of all proto-oncogenes [6,7]. It has a central regulatory role in a variety of cancer cell processes, including growth, adhesion, motility (migration/invasion), survival, and metastasis [7,8,9,10]. SFKs are also known to be deregulated in aggressive cancers via upregulation of protein expression and/or hyperactivation. In vitro molecular studies have revealed a role for SFKs in mitosis, but their precise role in mitotic cells is not well studied. Early studies revealed that active Src is required in mitosis [11,12]. Additionally, Src inhibition caused mitotic arrest [13], and Src activity is increased following entry into mitosis via CDK1 phosphorylation. More recently, it has also been reported that Src also has a critical role in cytokinesis, and this role is at least partially mediated through MAPK/ERK activation [14,15]. Generally speaking, the role of SFK activation in mitotic entry and exit is known. Far less is known regarding the role of Src during mitosis. It has been reported that c-Src kinase is associated with mitotic spindles and spindle poles in mouse cells [16]. In advanced PCa cells, c-Src activity is critical in spindle microtubule formation, and proper mitotic spindle orientation/structure entering metaphase [17]. Thus, it is critical to identify Src substrates in mitosis, which could shed light on the precise mechanisms of Src-mediated mitotic spindle regulation.

TUBB3 (Class III β-tubulin, βIII-tubulin) is one TUBB isoform of particular interest as a possible Src substrate. Under normal physiological conditions, TUBB3 is most abundantly expressed in neural and neuroendocrine tissues (neurons, testicular sertoli cells), but it is also expressed transiently or at basal levels in other normal tissues [18]. It is a critical component of mitotic spindles in a wide range of human cells [19]. Microtubules enriched in TUBB3 are considerably more dynamic than those composed of other beta-tubulin isotypes. Increased expression of TUBB3 in cancer cells has emerged as a consistent biomarker of both (1) poor prognosis and (2) eventual taxane (paclitaxel and docetaxel) resistance in a wide variety of solid cancers, including breast cancer, non-small-cell lung cancer (NSCLC), and prostate cancer [20,21,22,23,24,25,26,27]. TUBB3 is able to form complexes with gamma-tubulin in vivo, which has a regulatory role in polar orientation and assembly of microtubules [28,29]. Additionally, TUBB3 expression is precisely regulated in a cell cycle-dependent manner and can alter the binding of tubulin-targeted drugs to microtubules [30,31]. In NSCLC cells, knockdown of TUBB3 enhanced the cytotoxic effects of paclitaxel and cisplatin. Rescue of TUBB3 expression restored anchorage-independent growth and enhanced the effects of both drugs [32,33,34]. More recent studies in NSCLC and breast cancer cells have revealed a very interesting dose-dependent divergence in response to DTX, based on the presence or absence of TUBB3. This presents the very intriguing possibility of TUBB3 functioning not only within the confines of the microtubule network but also independently as a cellular signaling factor [35]. Taken together, these data represent major advances in our understanding of TUBB3 upregulation and its role in the therapeutic response, but the precise molecular mechanisms that regulate TUBB3 activity in vivo are still unclear.

Microtubules, the target of taxane compounds, are dynamic polymers of repeating α-/β-tubulin heterodimers. They have a central role in cell growth, survival, motility, and mitosis [36,37,38,39]. Post-translational modifications (PTMs) are a ubiquitous mechanism of microtubule regulation. Various β-tubulin PTMs have been characterized over the last 20 years (including glutamylation, detyrosination, acetylation, palmitoylation, and phosphorylation), but we are now only beginning to unravel the complexities of the interplay among β-tubulin PTMs and their roles in both normal physiology and disease [39,40]. Src-mediated tyrosine phosphorylation of β-tubulin has been observed in developing neurons [41] and in HL-60 leukemia cells [42]. Unfortunately, very little else is known about the role of tyrosine phosphorylation in the regulation of β-tubulin, especially in the context of advanced cancer [43]. More specifically, Src-mediated modulation of microtubules (and more specifically, β-tubulin) has remained almost completely unexplored, especially in the context of solid malignancies [9,10]. Interestingly, it has been reported that inhibition of SFKs can enhance taxane sensitivity in ovarian cancer cell lines, suggesting a link between SFK-mediated signaling and resistance to tubulin-targeting drugs [44,45]. Other Src kinases, including Fyn and Lyn kinases, have also previously been shown to associate with tubulins in mouse oocytes [46,47]. 

In the current study, we characterized the regulation of β-tubulin isoform TUBB3 by SRC-mediated tyrosine phosphorylation and its role in the regulation of subcellular localization and protein stability of TUBB3 and mitotic spindle stability in prostate cancer cells.

## 2. Materials and Methods

### 2.1. Cell Lines and Cell Culture 

CWR-R1 cells were a generous gift from Drs. Gregory and Wilson of the University of North Carolina Chapel Hill. R1/DTX is the Docetaxel-resistant derivative of CWR-R1 described previously [48] All other cell lines were initially purchased from American Tissue Culture Collections (ATCC). LNCaP, C42B, PC3, and DU145 cell lines were cultured in Corning Cellgro RPMI medium supplemented with 10% FBS and 1% pen/strep (p/s). CWR-22Rv1, CWR-R1, and R1/DTX cell lines were cultured in Corning Cellgro RPMI medium supplemented with 10% Heat-inactivated (HI) FBS and 1% p/s. Heat inactivation of FBS is achieved by incubating in 37 °C water bath for 1 h. COS-1 and HEK-293T cells were cultured in Dulbecco’s Modification of Eagle’s Medium (DMEM) supplemented with 10%FBS and 1% p/s.

### 2.2. Cloning and Constructs 

Human TUBB3 cDNA was subcloned into pcDNA3.1 or pEGFP-C2, as previously described [49]. All human GFP-TUBB3 constructs have a C-terminal GFP tag. Tyrosine site mutants were generated using Stratagene Quickchange Mutagenesis Kit. All mutant sites were confirmed by sequencing through University of Maryland Genomics Core Facility. The shRNAs specific for human TUBB3 were purchased from Sigma. 

### 2.3. Transient Transfections 

COS-1 cells were plated into 6-well dishes and allowed to grow for 24 h. Cells were then transfected using Xtremegene HP transfection reagent (Roche), using 2:1 reagent:plasmid ratio. Cell lysates were collected 48 h post-transfection.

### 2.4. Cell Viability Assays 

Cell viability was measured using a CCK-8 assay (VitaScientific, Beltsville, MD, USA). Cells were seeded in 96-well plates (Thermo Fisher Scientific, Waltham, MA, USA) at a density of 2000 cells/well and co-infected with indicated lentiviruses for 24 h, then allowed to grow in 1% heat-inactivated FBS supplemented with either DMSO or Docetaxel. After 7 days, cells were incubated with CCK-8 reagent (10 µL/well) for 2 h. Absorbance was measured using a plate reader at 450 nm wavelength. 

### 2.5. Immunoprecipitation (IP), Western Blots, and Antibodies 

For immunoprecipitations (IPs) and Western Blots, cell lysates were collected using Radioimmunoprecipitation (RIPA) buffer supplemented with inhibitors (PVDF, NaVO4, Aprotinin, NaF). Cell lysates were then subjected to 30 s sonication at 10% amplitude, and centrifuged at 4 °C for 15 min. Protein concentrations of the cleared lysates were determined using Bio-Rad DC Protein Assay. An aliquot of lysates was mixed with equal volume of 2X SDS sample buffer. Western Blot and IP were carried out as previously described [50]. For IP, cell lysates were incubated with ~1 μL antibody per 1 mL lysate at 4 °C for ~16 h. For reciprocal IP, cell lysates were collected from p100 plates using 1 mL of RIPA. 100 μL were then taken for total cell lysate (TCL, 10% input), and remaining lysate was split evenly into two 400 μL aliquots for IP with indicated antibodies. For Western Blot analyses, lysates were separated on 8–10% SDS-Polyacrylamide gels. Proteins were transferred to Immobilon-P PVDF membranes (Millipore). Membranes were blocked using 5% milk or 3% BSA, then incubated at 4 °C overnight in primary antibody. TUBB3 antibodies used: Mouse monoclonal TUBB3 (TUJ1) (Covance MMS-435p, 1:10,000), Mouse monoclonal TUBB3 (Chemicon TU20, 1:800), Rabbit polyclonal TUBB3 (Abcam, 1:10,000). Other antibodies include anti-pSFK-Y416 (Cell Signaling, 1:1000), anti-alpha-tubulin (Sigma, 1:10,000), anti-phosphotyrosine (Santa Cruz PY99, 1:1000), anti-tubulin (ABM G098, 1:10,000), anti-GAPDH (Santa Cruz, 1:5000) anti-HSP90 (Santa Cruz, 1:10,000), and anti-PARP1 (Santa Cruz, 1:1000). The pTUBB3-Y340 antibody was developed by immunizing rabbits with a synthetic phospho-peptide corresponding to phosphorylated Tyrosine 340 of TUBB3, followed by two-round affinity purification using immunogen (NeoBioLab). Densitometry quantification was conducted using image J densitometry function using publicly available protocol (http://stanxterm.aecom.yu.edu/wiki/index.php?page=Using_ImageJ, accessed on 15 August 2015).

### 2.6. Subcellular Fractionation 

Cells were grown in Thermo p100 dishes and treated with either vehicle or 10 μM SU6656 for 2 h before lysis. Cell lysis and fractionation were carried out according to manufacturer’s protocol (Pierce NE-PER nucleocytoplasmic fractionation kit).

### 2.7. Immunofluorescence (IF) and Quantification 

Cells were grown on glass coverslips, treated as indicated in figure legends, then fixed using 3.7% PFA. Once fixed, staining proceeded as previously described [51]. Epi-fluorescent imaging was carried out on a Nikon TE2000-U inverted microscope system, and NIS Elements acquisition software. Both 60x and 100x oil objectives were used. All confocal imaging was carried out on a Zeiss LSM-510 microscope using 60x oil objective. Quantification was completed by manual cell count. For each slide, at least 100 total cells were counted. The number of cells with intact mitotic spindles was determined by counting cells with two visible centrosomes at the spindle poles, condensed chromosomes lined up in the middle of the mitotic spindle (along metaphase plate) and organized microtubules between the centrosomes and the metaphase plate.

### 2.8. Protein Stability Assays 

COS-1 Cells were plated into 6-well plates and transfected as indicated. Cells were then treated with 100 μg/mL CHX for indicated time points. Lysates were then collected as described. For endogenous protein stability, prostate cancer cells were plated into 6-well plates and allowed to grow for 72 h. Before CHX treatment, cells were pre-treated for 2 h with either vehicle (DMSO) or SU6656, then treated with CHX for indicated time. All MG132 treatments were conducted at 50 μM concentration.

### 2.9. Phosphorylation Predictive Software, and 3-D Molecular Modeling 

NetPhos 2.0 software (http://www.cbs.dtu.dk/services/NetPhos/ accessed on 15 August 2015) was used to identify putative tyrosine phosphorylation sites of TUBB3. All 3D modeling was conducted using publicly available Mac PyMol 1.5 software and TUBA/TUBB dimer structures (PDB# 1JFF and PDB 6S8L).

## 3. Results

### 3.1. Tyrosine Phosphorylation of TUBB3 Mediated by SRC Kinase in PCa Cells

**Figure 1:**
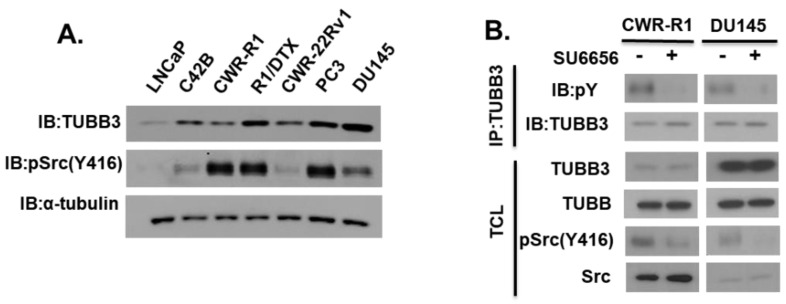
Expression and tyrosine phosphorylation of TUBB3 in prostate cancer cell lines. (**A**) Cell lysates from various PCa cell lines were subjected to immunoblotting using the indicated antibodies. (**B**) PCa cells were treated with either vehicle DMSO or 10 μM SU6656 (Src-inhibitor) for 2 h. Cell lysates were then subjected to immunoprecipitation (IP) with anti-TUBB3 (TUJ1) antibody, and blotted with indicated antibodies. Total cell lysates (TCL, 10% input) were also collected before IP and subjected to immunoblotting with indicated antibodies.

We examined TUBB3 expression in a panel of PCa cell lines (Figure 1A). Interestingly, we observed a significant increase in TUBB3 expression from the androgen-sensitive LNCaP cell line to its androgen insensitive isogenic C42B derivative isolated from bone metastasis (Lane 1 cf. Lane 2). We also observed an expected increase in TUBB3 expression when comparing the castration-resistant CWR-R1 cell line to its isogenic DTX-resistant derivative R1/DTX cell line (Lane 3 cf. Lane 4). Finally, the more aggressive/metastatic cell lines (PC3 and DU145) showed a higher expression of TUBB3. We also examined SRC kinase activation status and found it was also elevated in a majority of the advanced PCa cell lines (R1, R1/DTX, PC3, DU145) compared to less aggressive LNCaP cells. We wonder whether TUBB3 is tyrosine phosphorylated in PCa cells and whether SRC kinase may modulate TUBB3 phosphorylation. To test this hypothesis, we treated CWR-R1 and DU145 cells with a selective SFK inhibitor SU6656 and examined the tyrosine phosphorylation status of TUBB3 using an anti-pY antibody. As shown in Figure 1B, TUBB3 was phosphorylated at tyrosine in both cell lines, and this tyrosine phosphorylation decreased following Src-inhibition by SU6656.

### 3.2. Identification of TUBB3 Phosphorylation Site 

**Figure 2:**
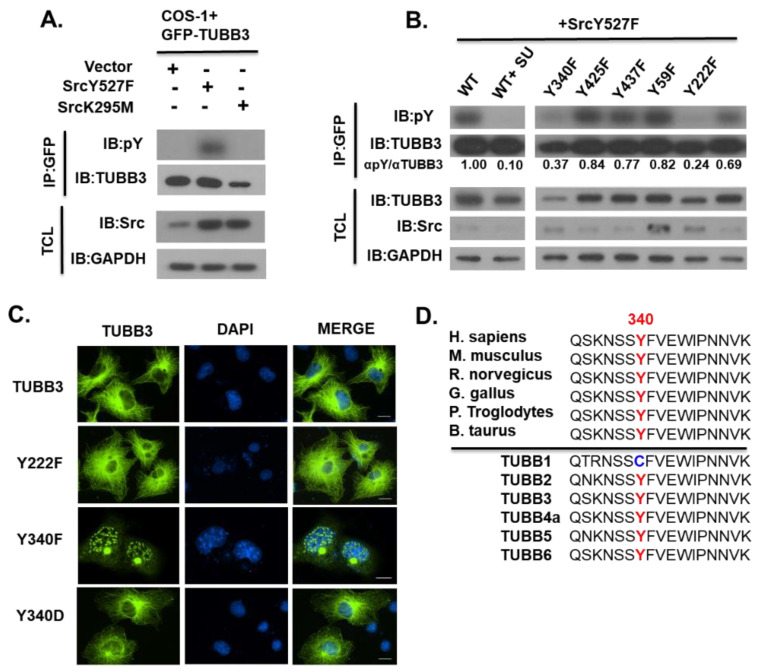
Identification of TUBB3 phosphorylation site. (**A**) COS-1 cells were transiently co-transfected with GFP-TUBB3 and either Empty Vector, constitutively active Src kinase (Y527F), or kinase-dead Src kinase (K295M). Cell lysates were then subjected to IP with anti-GFP antibody and blotted along with TCL with indicated antibodies. (**B**) COS-1 cells were transiently co-transfected with SrcY527F and GFP-TUBB3 (WT or Y-F mutant as indicated). Lysates were collected at 48 h post-transfection, subjected to Immunoprecipitation with anti-GFP antibody, and blotted along with TCL for indicated antibodies. (**C**) COS-1 Cells were plated onto glass coverslips and transiently transfected with indicated untagged constructs at 24 h post-plating. Cells were fixed at 48 h post-transfection and stained using mouse anti-TUBB3 (TUJ1, Green) and DAPI (Blue). Image magnification for all is 60x. Scale bar represents 14 μm. (**D**) Multiple-sequence alignment of TUBB3 protein among species (Top panel) and Human TUBB isoforms (Bottom panel). Residue 340 is a cysteine in the ubiquitously expressed TUBB1 but is a conserved tyrosine in all other TUBB isoforms.

We then set out to further investigate a possible role of Src kinase in TUBB3 tyrosine phosphorylation. First, we observed a robust increase of tyrosine phosphorylation of GFP-TUBB3 when co-expressed with the constitutively active Src (Y527F), and this phosphorylation was significantly decreased when it was co-expressed kinase-dead Src(K295M) (Figure 2A), suggesting the active Src kinase can induce tyrosine phosphorylation of TUBB3. To determine putative TUBB3 tyrosine phosphorylation sites, we first used NetPhos 2.0 phosphorylation prediction software to analyze the TUBB3 protein sequence (Accession Number: Q13509). Prediction software analysis suggested six putative phosphorylation sites. We then mutated these tyrosine residues individually and tested the effects on SRC-induced phosphorylation. As shown in Figure 2B mutation of Y222 or Y340 significantly diminished tyrosine phosphorylation, while mutation of the other four tyrosine residues (Y59, Y281, Y435, and Y437) did not alter TUBB3 phosphorylation. These data suggested that Y222 and Y340 could be the major phosphorylation sites induced by SRC kinase. We also examined the subcellular localization of these two mutants and found that the phospho-deficient Y340F mutant displayed a drastically different localization pattern, with predominantly nuclear staining and perinuclear accumulation in a region, which we postulated to be the centrosome (Figure 2C). The phosphomimetic Y340D mutant rescued compromised microtubule localization. Meanwhile, the Y222F mutant mainly localized into the microtubule network as the wild-type TUBB3 did. We also used the publicly available ClustalOmega webserver to align TUBB3 sequences from various species and all human TUBB isoform sequences for comparison. Interestingly, Y340 is highly conserved in TUBB3 among species (Figure 2D, upper) and all TUBB isoforms, except for TUBB1 (Figure 2D, lower). The corresponding residue for TUBB3 Y340 is a cysteine in TUBB1, suggesting that modification of Y430 may confer a selective regulation of TUBB3 that is not applicable to the ubiquitously expressed TUBB1. Taken together, our results suggest that Y340 is one of the major phosphorylation sites induced by Src and may modulate TUBB3 subcellular localization and is critical for proper assembly of TUBB3 into microtubules. 

### 3.3. Modulation of TUBB3 Y340 Phosphorylation by SRC Kinase in Response to EGF and Docetaxel

**Figure 3:**
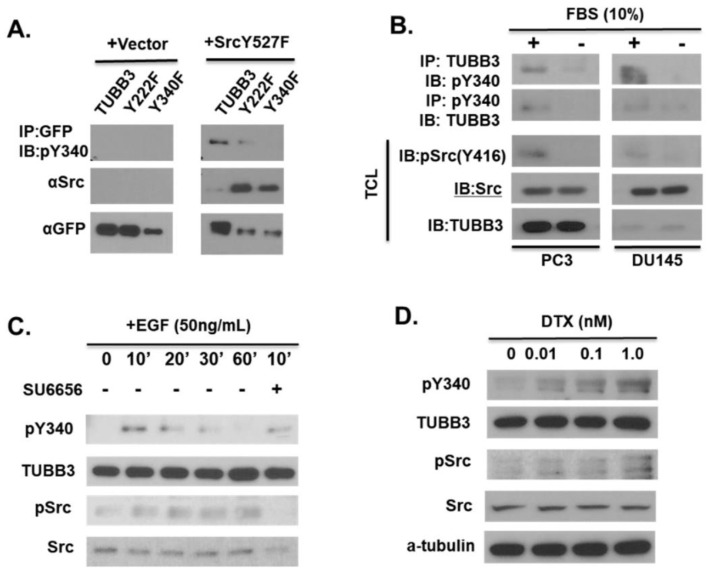
Modulation of TUBB3 Y340 phosphorylation by SRC kinase in response to EGF and docetaxel. (**A**) COS-1 cells were transiently transfected with GFP-TUBB3, GFP-TUBB3-Y222F, or GFP-TUBB3-Y340F, in the presence or absence of constitutively active Src. Lysates were collected 48 h post-transfection and subjected to IP with anti-GFP antibody. IP lysate and total cell lysate were blotted with indicated antibodies. (**B**) DU145 and PC3 cells were serum-starved for 24 h before lysis. Lysates were then split into equal aliquots, subjected to reciprocal IP with mouse anti-TUBB3 (TUJ1) or rabbit anti-pTUBB3-Y340, then blotted along with TCL for indicated antibodies. (**C**) CWR-R1 CRPC cells were serum-starved for 24 h, then treated with 50 ng/mL Epidermal Growth Factor (EGF) for indicated times. The maximum pY340 phosphorylation was observed at 10′, which was blocked by simultaneous treatment with SU6656. (**D**) DU145 cells were treated with indicated concentrations of docetaxel (DTX) for 24 h and cell lysates were subjected to immunoblotting with indicated antibodies.

To further characterize Y340 phosphorylation, we developed a phospho-specific antibody for Y340 of TUBB3 (pY340). This antibody is capable of detecting Src-induced phosphorylation of GFP-fused wild-type TUBB3 or the Y222F mutant but failed to detect phosphorylation of the Y340F mutant (Figure 3A), confirming the specificity of our antibody. To determine what stimuli could induce TUBB3 Y340 phosphorylation, we examined Y340 phosphorylation of endogenous TUBB3 in PCa cells using reciprocal immunoprecipitation (IP) under various conditions. We detected increased Y340 phosphorylation in both DU145 and PC3 cells in response to serum (Figure 3B). Given that there is a myriad of signaling molecules in full serum, we next set out to examine the effect of a more specific stimulus of Src kinase activation. In previous studies, we found that EGF treatment enhances Src activation in LNCaP cells [51]. Treatment with 50 ng/mL EGF was sufficient to induce Y340 phosphorylation at the 10-minute time point (Lane 2, Figure 3C), and this phosphorylation at 10 min was strongly inhibited by simultaneous treatment with SU6656 (Lane 6, Figure 3C). Finally, we investigated the effect of docetaxel treatment on the TUBB3 pY340 signal. Interestingly, we found that DTX treatment was sufficient to enhance TUBB3 pY340, and this phosphorylation increased with an increasing dose of DTX (Figure 3D).

### 3.4. Phosphorylation of Y340 Stabilizes TUBB3 Protein

**Figure 4:**
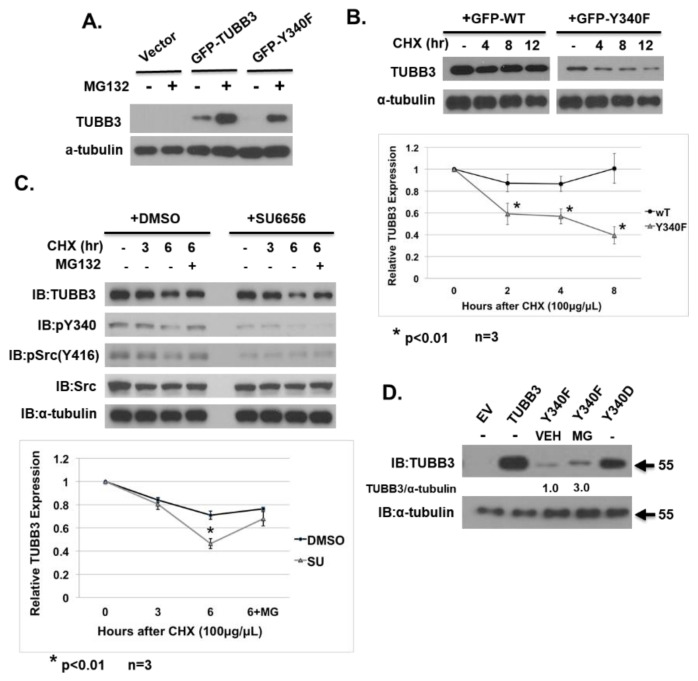
Phosphorylation of Y340 stabilizes TUBB3 protein. (**A**) COS-1 cells were transiently transfected with Empty Vector, GFP-TUBB3, or GFP-TUBB-Y340F. Cells were allowed to grow for 48 h post-transfection, then treated with either vehicle (DMSO) or proteasome inhibitor MG132. Cell lysates were subjected to immunoblotting with indicated antibodies. (**B**) Top panel, COS-1 cells were transiently transfected 24 h post-plating with either GFP-TUBB3 or GFP-Y340F. At 48 h post-transfection, cells were treated with cycloheximide (CHX) for indicated times. Cell lysates were subjected to immunoblotting with indicated antibodies. Bottom panel, densitometry quantification TUBB3/alpha-tubulin ratio of the top panel. (**C**) Top panel, R1/DTX cells were pretreated for 2 h with either vehicle or SU6656, then treated with CHX, or combined CHX/MG132 for indicated time points. Cell lysates were subjected to immunoblotting with indicated antibodies. Bottom panel, densitometry quantification of TUBB3/alpha-tubulin ratio of the top panel. (**D**) HEK-293T cells were transiently transfected with untagged human TUBB3 constructs (empty vector (EV), Wild-type (WT), Y340D, Y340F). At 48 h post-transfection, cells were treated with DMSO(VEH) or MG132(MG) for 24 h before cells were lysed. Cell lysates were then subjected to immunoblotting with indicated antibodies.

Regulation of TUBB3 expression by proteasome degradation has been previously reported [43]. During previous experiments, we consistently observed a sharp decrease in overall GFP-Y340F expression level when compared to GFP-WT (Figure 2B). We suspected that this could possibly be due to the destabilization of the protein. We first found that the decreased expression of GFP-Y340F could be rescued to detectable levels by treatment with a proteasome inhibitor MG132 (Figure 4A). Additionally, we found that GFP-Y340F half-life was significantly decreased when compared to GFP-WT (Figure 4B). Furthermore, the half-life of endogenous TUBB3 is dramatically reduced following 2 h pre-treatment with Src inhibitor, but protein expression was rescued by MG132 treatment (Figure 4C). Finally, transient transfection of 293T cells with untagged human TUBB3 constructs revealed that TUBB3 Y340F expression could be rescued by MG132 treatment (Figure 4D, lane 6). Taken together, these data indicate that TUBB3 Y340 phosphorylation can be modulated by Src kinase activation or inhibition, and phosphorylation of Y340 stabilizes TUBB3 protein.

### 3.5. Effects of Y340 Phosphorylation on TUBB3 Subcellular Localization and Mitotic Spindle Stability

**Figure 5:**
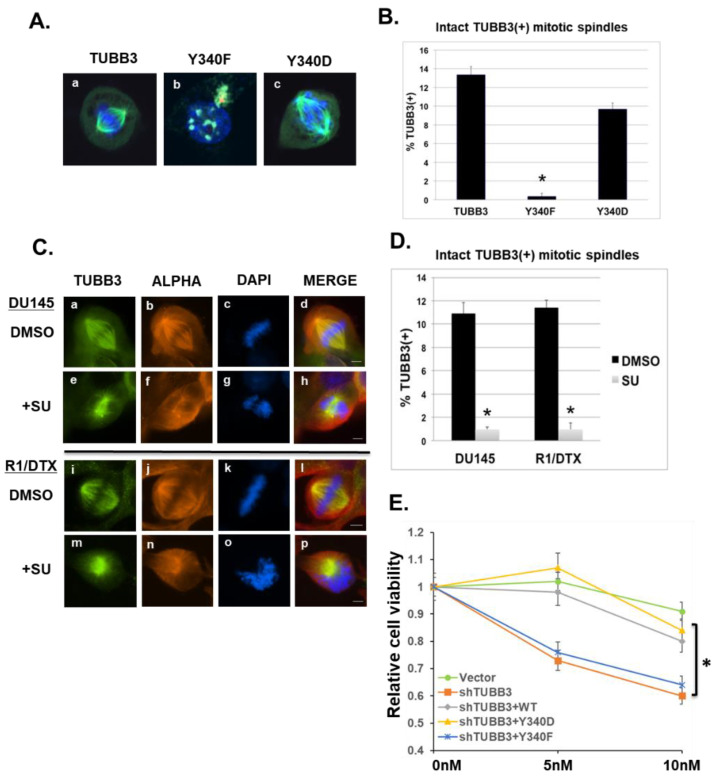
Effects of TUBB3 Y340 phosphorylation on its subcellular localization and mitotic spindle stability and drug sensitivity. (**A**) COS-1 cells were transiently transfected 24 h post-plating with indicated plasmids (TUBB3, Y340F, Y340D). Cells were allowed to grow for an additional 48 h on glass coverslips before collection, fixation, and co-stain with mouse monoclonal anti-TUBB3 (green), rabbit anti-pericentrin (red), and DAPI (blue, for DNA). Images were taken using a Zeiss LSM510 Laser Scan Confocal Microscope. (**B**) Quantification of cell count from 3 independent repeats of experiments shown in Panel (A). All cell counts were conducted by visual inspection. At least 100 total cells were counted for each condition. * *p* < 0.01. (**C**) DU145 cells (a–h) OR docetaxel-resistant R1/DTX cells (i–p) were grown on glass coverslips for 48 h, then treated for 2 h with either DMSO (a–d, i–l) or Src-inhibitor SU6656 (e–h, m–p). Cells were then co-stained with mouse anti-alpha-tubulin (red), rabbit anti-TUBB3 (green), and DAPI (blue). Scale bar represents 4.5 μm. (**D**) Slides from left panels were then quantified by cell counting for intact, bipolar, TUBB3-positive mitotic spindles. At least 100 total cells were counted in each experimental replicate. * *p* < 0.01. (**E**) R1-DTX Cells were co-infected with lentivirus encoding shRNA specific for TUBB3 (shTUBB3) with Vector control or the indicated TUBB3 or mutants for 24 h. Cells were then allowed to grow in 1% heat-inactivated FBS supplemented with either DMSO or Docetaxel (5 nM or 10 nM) for 7 days. Cell viability was determined using CCK-8 assays. The value of each DMSO control (0 nM DTX) was set as 1. The relative cell viability was expressed as the ratio between DTX-treated and DMSO-treated cells. * *p* < 0.01.

Suppression of microtubule dynamics, or mitotic slippage, is a major mechanism of taxane-mediated cell death [37,43]. It has been previously reported that Src is a direct regulator of mitotic spindles [17]. It is also known that TUBB3 is a critical component of mitotic spindle microtubules (both astral and kinetochore microtubules) in mammalian cells [19] and that removal of TUBB3 from the total tubulin pool significantly increases in vitro microtubule assembly. Recently, Ertych et al. demonstrated that increased spindle microtubule assembly directly contributes to chromosomal instability and in vivo tumor progression in colorectal cancer cells [52]. Consistent with previous reports, we found that exogenous TUBB3 localizes strongly into mitotic spindles (Figure 5A, panel a), as did the phosphomimetic Y340D mutant (Figure 5A, panel c). However, the phosphodeficient TUBB3-Y340F mutant (shown in an interphase cell in Figure 5A, panel b) was unable to incorporate into mitotic spindles. Quantification of intact TUBB3-positive mitotic spindles (Figure 5B) revealed a significant loss in Y340F-expressing cells and a concomitant rescue of activity in Y340D-expressing cells. Unsurprisingly, we also observed a sharp decrease in the number of intact bipolar TUBB3(+) spindles following SU6656 treatment of highly aggressive DU145 cells, and taxane-resistant R1/DTX cells (Figure 5C). These SU6656-treated mitotic cells show complete abrogation of proper metaphase plate structure and chromosome orientation (Figure 5C, e–h/m–p). Quantification by cell count (Figure 5D) revealed a highly significant reduction in visibly intact TUBB3(+) mitotic spindles in both cell lines. These data suggest that SU6656 treatment of PCa cell lines dramatically inhibited mitotic spindle dynamics and resulted in disorganized chromosome distribution and compromise of mitotic spindle architecture. Furthermore, when the endogenous TUBB3 in drug-resistant R1-DTX cells was knockdown by a specific shRNA, cells became more sensitive to docetaxel (Figure 5E). While both WT and the phosphomimetic Y340D mutant could rescue the loss of TUBB3, the phosphodeficient Y340F mutant failed to do so. Our results suggest that TUBB3 Y340 phosphorylation is critical for TUBB3 mediated drug resistance possibly through modulating its stability and incorporation into microtubules. 

**Figure 6:**
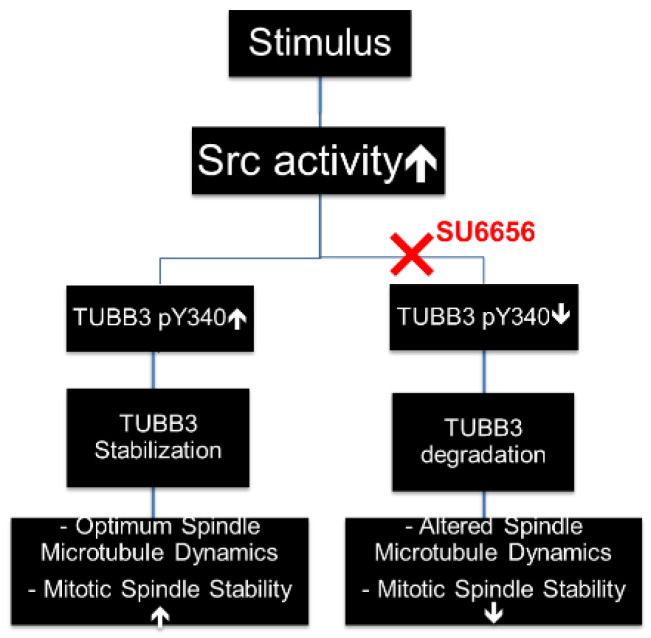
Working model of regulation of TUBB3 by SRC kinase. Upstream stimuli activate SRC kinase, resulting in an increase of TUBB3 phosphorylation at Y340. This increased Y340 phosphorylation results in enhanced TUBB3 stability, which leads to mitotic spindle stability, TUBB3-mediated cell survival, and decreased effectiveness of DTX treatment. However, pharmacological inhibition of SRC kinase, such as with SU6656, decreases TUBB3 Y340 phosphorylation and reduces stability of TUBB3, which in turn leads to compromised mitotic spindle integrity in dividing cells.

In summary, our data indicate that TUBB3 is a novel downstream target of Src-mediated tyrosine phosphorylation. Phosphorylation of tyrosine 340 stabilizes TUBB3 protein and also facilitates TUBB3-mediated stabilization of mitotic spindles in dividing cells (Figure 6).

## 4. Discussion

It is well known that both SFK and TUBB3 play critical roles in mitosis. Deregulation of TUBB3 in tumorigenicity and tumor progression are often coupled with the prevalence of SFK dysfunction in a similar spectrum of cancers. However, very little has been characterized regarding their possible interplay in regulating mitotic spindle dynamics and drug sensitivity. In this report, we have shown that TUBB3 expression is significantly increased during PCa progression using several isogenic cell systems. Firstly, TUBB3 expression is increased in castration-resistant C4-2B cells compared to isogenic parental LNCaP cells. Secondly, we found that TUBB3 is increased in 10 nM DTX-resistant R1/DTX cells compared to isogenic parental CWR-R1 cells. These findings support previous studies that have suggested TUBB3 to have a key role in the transition to castration resistance, and also a key role in the further transition to taxane resistance. The molecular mechanisms by which TUBB3 mediates these transitions are not well characterized, thus warranting further studies. Furthermore, we report here for the first time that TUBB3 is phosphorylated at tyrosine by active Src kinase in prostate cancer cells, specifically at tyrosine 340 (Y340). We found that this Y340 phosphorylation is dramatically decreased upon Src inhibition with chemical inhibitors such as SU6656, or concomitantly increased following Src activation with mitogenic media containing FBS. Y340 phosphorylation (and, therefore, TUBB3 protein stability) is also markedly increased by EGF, given the widely reported role of this particular growth factor in the persistence and aggression of advanced PCa. Functionally, we found that loss of phosphorylation at Y340 significantly reduces TUBB3 protein half-life, and has a marked effect on the architecture of mitotic spindles. Taken together, these data indicate that phosphorylation of tyrosine 340, stabilizes TUBB3 protein and also facilitates TUBB3-mediated stabilization of mitotic spindles in dividing cells. Our findings are summarized in Figure 6. 

Through mutagenesis and structural studies, we also found that Y340 is one of the major phosphorylation sites induced by Src kinase, despite the lack of a canonical Src substrate consensus sequence. Microscopy experiments also revealed that Y340F mutant localized to punctae inside the cell nucleus, and also aggregated at the centrosome of interphase cells (Figure 2C and Figure 5A [panel b]). This was very interesting, as a non-microtubule role of TUBB3 as a sub-cellular survival factor has been suggested previously [37]. Given that exogenous Y340F mutant localized into the nucleus (Figure 2C and Figure 5A), and that SU6656 treatment strongly altered mitotic spindle stability of PCa cells. Further studies will be needed in the future to determine the functional significance of TUBB3 nuclear translocation in taxane-resistant cancer cells.

Though we found Y340 to be a major SFK-mediated phosphorylation site in TUBB3, several other residues warrant further study based on their location on TUBB3 protein. These include Y222 (located directly over the GDP-binding pocket), Y281 (located in the critical TUBB M-loop), Y425 (specific for TUBB3 and TUBB4), and Y437 (TUBB3 specific, and located in a highly acidic region). Comprehensive studies are required to further characterize the role of each site in the regulation of TUBB3 activity, localization, and binding partners. It is also critical to characterize the interplay of TUBB3 phosphorylation sites with other TUBB modifications (such as acetylation, methylation, de-tyrosination, and Serine/Threonine phosphorylation). The current study mainly focused on the effects of Src on TUBB3 phosphorylation. Y340 is also conserved in other beta tubulins (except for TUBB1). Future research is needed to investigate whether Y340 phosphorylation plays a role in regulating the physiological activity of other beta tubulins. 

Current treatments involving DTX and/or Src-inhibitors produce a number of deleterious side effects in patients. Thus, we must continue searching for safer and more specific chemotherapeutic options for advanced cancer. Elucidating molecular mechanisms of TUBB3 regulation in cancer cells could lead to the improvement of existing microtubule-targeted therapies, or also to the development of effective specific TUBB3-targeted therapies. Given that (1) TUBB3 expression and (2) dysfunction of SFKs both are associated with poor outcomes in a variety of solid cancers, a deeper understanding of the interplay between SFKs and β-tubulin in advanced prostate cancer could also improve clinical treatment of other solid cancers, including breast cancer and NSCLC. Furthermore, it is plausible that the phosphorylation status of TUBB3 could be a predictive biomarker of prognosis or drug response. This could be especially applicable to prostate cancer. Although Dasatinib (Src-inhibitor) and taxane compounds have proven to be effective in combination therapy of other advanced cancers, such as NSCLC, the phase III clinical trial concluded that Dasatinib (Src inhibitor) failed to improve patient outcomes when combined with Docetaxel/Prednisone in advanced CRPC patients. Given that patients were randomly selected in the trial and a subpopulation of patients did respond favorably, it would be interesting to examine whether the level of TUBB pY340 could predict patients’ response to the combination therapy. Thus, it is possible that TUBB/Src regulatory interaction could conceivably be a single point of therapeutic intervention in the future. Further study is needed, however, before these critical clinical challenges can be overcome. 

## Data Availability

Not applicable.

## References

[1] (2013). Cancer Facts & Figures 2013. American Cancer Society Website. https://www.cancer.org/research/cancer-facts-statistics/all-cancer-facts-figures/cancer-facts-figures-2013.html.

[2] Shen M., Shen C. (2010). Molecular Genetics of Prostate Cancer. Genes Dev..

[3] George D., Moul J. (2012). Emerging Treatment Options for Patients with Castration-Resistant Prostate Cancer. Prostate.

[4] Shapiro D., Tareen B. (2012). Current and Emerging Treatments in the Management of Castration-Resistant Prostate Cancer. Expert Rev. Anticancer Ther..

[5] Kim L.C., Song L., Haura E.B. (2009). Src kinases as therapeutic targets for cancer. Nat. Rev. Clin. Oncol..

[6] Stehelin D., Varmus H.E., Bishop J.M., Vogt P.K. (1976). DNA related to the transforming gene(s) of avian sarcoma viruses is present in normal avian DNA. Nature.

[7] Yeatman T.J. (2004). A Renaissance for SRC. Nat. Rev. Cancer.

[8] Chang Y.M., Kung H.J., Evans C.P. (2007). Nonreceptor Tyrosine Kinases in Prostate Cancer. Neoplasia.

[9] Fizazi K. (2007). The role of Src in prostate cancer. Ann. Oncol..

[10] Guarino M. (2010). Src Signaing in Cancer Invasion. J. Cell. Physiol..

[11] Roche S., Fumagalli S., Courtneidge S.A. (1995). Requirement for Src family protein-tyrosine kinases in G2 for fibroblast cell division. Science.

[12] Chackalaparampil I., Shalloway D. (1988). Altered phosphorylation and activation of pp60c-Src during fibroblast mitosis. Cell.

[13] Moasser M.M., Srethapakdi M., Sachar K.S., Kraker A.J., Rosen N. (1999). Inhibition of Src kinases by a selective inhibitor causes mitotic arrest. Cancer Res..

[14] Ng M.M., Chang F., Burgess D.R. (2005). Movement of membrane domains and requirement of membrane signaling molecules for cytokinesis. Dev. Cell.

[15] Kasahara K., Nakayama Y., Nakazato Y., Ikeda K., Kuga T., Yamaguchi N. (2007). Src signaling regulates completion of abscission in cytokinesis through ERK/MAPK activation at the midbody. J. Biol. Chem..

[16] David-Pfeuty T., Bagrodia S., Shalloway D. (1993). Differential localization patterns of myristoylated and non-myristoylated c-Src proteins in interphase and mitotic c-Src overexpressor cells. J. Cell Biol..

[17] Nakayama Y., Matsui Y., Takeda Y., Okamoto M., Abe K., Fukumoto Y., Yamaguchi N. (2012). c-Src but not Fyn Promotes Proper Spindle Orientation in Early Prometaphase. J. Biol. Chem..

[18] Kilpinen S., Autio R., Ojala K., Iljin K., Bucher E., Sara H., Pisto T., Saarela M., Skotheim R.I., Björkman M. (2008). Systematic bioinformatics analysis of expression levels of 17,330 human genes across 9,783 samples from 175 types of healthy and pathological tissues. Genome Biol..

[19] Jouhilahti E.M., Peltonen S., Peltonen J. (2008). Class III β-tubulin Is a Component of the Mitotic Spindle in Multiple Cell Types. J. Histochem. Cytochem..

[20] Tommasi S., Mangia A., Lacalamita R., Bellizzi A., Fedele V., Chiriatti A., Thomssen C., Kendzierski N., Latorre A., Lorusso V. (2007). Cytoskeleton and paclitaxel sensitivity in breast cancer: The role of β-tubulins. Int. J. Cancer.

[21] Hetland T.E., Hellesylt E., Flørenes V.A., Tropé C., Davidson B., Kærn J. (2010). Class III β-tubulin expression in advanced-stage serous ovarian carcinoma effusions is associated with poor survival and primary chemoresistance. Hum. Pathol..

[22] Vilmar A.C., Sontoni-Rugiu E., Sørensen J.B. (2011). Class III β-tubulin in Advanced NSCLC of Adenocarcinoma Subtype Predicts Superior Outcome in a Randomized Trial. Clin. Cancer Res..

[23] Hayashi Y., Kuriyama H., Umezu H., Tanaka J., Yoshimasu T., Furukawa T., Tanaka H., Kagamu H., Gejyo F., Yoshizawa H. (2009). Class III β-tubulin expression in Tumor Cells is Correlated with Resistance to Docetaxel in Patients with Completely Resected NSCLC. Intern. Med..

[24] Seve P., Mackey J., Isaac S., Trédan O., Souquet P.J., Pérol M., Lai R., Voloch A., Dumontet C. (2005). Class III β-tubulin expression in tumor cells predicts response and outcome in patients with NSCLC receiving paclitaxel. Mol. Cancer Ther..

[25] Terry S., Ploussard G., Allory Y., Nicolaiew N., Boissiere-Michot F., Maille P., Kheuang L., Coppolani E., Ali A., Bibeau F. (2009). Increased expression of class III β-tubulin in castration-resistant human prostate cancer. Br. J. Cancer.

[26] Ploussard G., Terry S., Maillé P., Allory Y., Sirab N., Kheuang L., Soyeux P., Nicolaiew N., Coppolani E., Paule B. (2010). Class III β-tubulin Expression Predicts Prostate Tumor Aggressiveness and Patient Response to Docetaxel-Based Chemotherapy. Cancer Res..

[27] Tsourlakis M.C., Weigand P., Grupp K., Kluth M., Steurer S., Schlomm T., Graefen M., Huland H., Salomon G., Steuber T. (2014). β(III)-tubulin is an independent predictor of prostate cancer progression tightly linked to ERG fusion status and PTEN deletion. Am. J. Pathol..

[28] Katsetos C.D., Dráberová E., Šmejkalová B., Reddy G., Bertrand L., de Chadarévian J.P., Legido A., Nissanov J., Baas P.W., Dráber P. (2007). Class III β-tubulin and gamma-tubulin are co-expressed and form complexes in human glioblastoma cells. Neurochem. Res..

[29] Oakley B.R. (2000). An abundance of tubulins. Trends Cell Biol..

[30] Shibazaki M., Maesawa C., Akasaka K., Kasai S., Yasuhira S., Kanno K., Nakayama I., Sugiyama T., Wakabayasi G., Masuda T. (2012). Transcriptional and post-transcriptional regulation of β(III)-tubulin protein expression in relation with cell cycle-dependent regulation of tumor cells. Int. J. Oncol..

[31] Narvi E., Jaakkola K., Winsel S., Oetken-Lindholm C., Halonen P., Kallio L., Kallio M.J. (2013). Altered TUBB3 expression contributes to the epothilone response of mitotic cells. Br. J. Cancer.

[32] McCarroll J.A., Gan P.P., Liu M., Kavallaris M. (2010). β(III)-tubulin is a Multifunctional Protein Involved in Drug Sensitivity and Tumorigenesis in Non-Small Cell Lung Cancer. Cancer Res..

[33] Kavallaris M., Kuo D.Y., Burkhart C.A., Regl D.L., Norris M.D., Haber M., Horwitz S.B. (1997). Taxol-resistant epithelial ovarian tumors are associated with altered expression of specific β-tubulin isotypes. J. Clin. Investig..

[34] Gan P.P., Pasquier E., Kavallaris M. (2007). Class III β-tubulin Mediates Sensitivity to Chemotherapeutic Drugs in Non-Small Cell Lung Cancer. Cancer Res..

[35] Gan P.P., McCarroll J.A., Po’uha S.T., Kamath K., Jordan M.A., Kavallaris M. (2010). Microtubule Dynamics, Mitotic Arrest, and Apoptosis: Drug-Induced Differential Effects of β(III)-tubulin. Mol. Cancer Ther..

[36] Verdier-Pinard P., Pasquier E., Xiao H., Burd B., Villard C., Lafitte D., Miller L.M., Angeletti R.H., Horwitz S.B., Braguer D. (2009). Tubulin proteomics: Towards breaking the code. Anal. Biochem..

[37] Kavallaris M. (2010). Microtubules and resistance to tubulin-binding agents. Nat. Rev. Cancer.

[38] Verhey K., Gaertig J. (2007). The Tubulin Code. Cell Cycle.

[39] Wloga D., Gaertig J. (2010). Post-translational modifications of microtubules. J. Cell Sci..

[40] Janke C. (2014). The tubulin code: Molecular components, readout mechanisms, and functions. J. Cell Biol..

[41] Matten W.T., Aubry M., West J. (1990). Tubulin is phosphorylated at tyrosine by pp60c-Src in nerve growth cone membranes. J. Cell Biol..

[42] Katagiri K., Katagiri T., Kajiyama K., Yamamoto T., Yoshida T. (1993). Tyrosine-Phosphorylation of Tubulin during Monocytic Differentiation of HL-60 cells. J. Immunol..

[43] Parker A.L., Kavallaris M., McCarroll J.A. (2014). Microtubules and their role in cellular stress in cancer. Front. Oncol..

[44] Chen T., Pengetnze Y., Taylor C.C. (2005). Src inhibition enhances paclitaxel cytotoxicity in ovarian cancer cells by caspase-9-independent activation of caspase-3. Mol. Cancer Ther..

[45] Le X.F., Bast R.C. (2011). Src family kinases and paclitaxel sensitivity. Cancer Biol. Ther..

[46] Levi M., Maro B., Shalgi R. (2010). The involvement of Fyn kinase in resumption of the first meiotic division in mouse oocytes. Cell Cycle.

[47] Levi M., Shalgi R. (2010). The role of Fyn kinase in the release from metaphase in mammalian oocytes. Mol. Cell. Endocrinol..

[48] Linn D.E., Yang X., Sun F., Xie Y., Chen H., Jiang R., Chen H., Chumsri S., Burger A.M., Qiu Y. (2010). A Role for OCT4 in Tumor Initiation of Drug-Resistant Prostate Cancer Cells. Genes Cancer.

[49] Xie Y., Xu K., Dai B., Guo Z., Jiang T., Chen H., Qiu Y. (2006). The 44kDa Pim-1 kinase directly interacts with tyrosine kinase Etk/BMX and protects human prostate cancer cells from apoptosis induced by chemotherapeutic drugs. Oncogene.

[50] Xie Y., Xu K., Linn D.E., Yang X., Guo Z., Shimelis H., Nakanishi T., Ross D.D., Chen H., Fazli L. (2008). The 44-kDa Pim-1 Kinase Phosphorylates BCRP/ABCG2 and Thereby Promotes Its Multimerization and Drug-resistant Activity. J. Biol. Chem..

[51] Yang X., Guo Z., Sun F., Li W., Alfano A., Shimelis H., Chen M., Brodie A.M., Chen H., Xiao Z. (2011). Novel Membrane-associated Androgen Receptor Splice Variant Potentiates Proliferative and Survival Responses in Prosate Cancer Cells. J. Biol. Chem..

[52] Ertych N., Stolz A., Stenzinger A., Weichert W., Kaulfuß S., Burfeind P., Aigner A., Wordeman L., Bastians H. (2014). Increased microtubule assembly rates influence chromosomal instability in colorectal cancer cells. Nat. Cell Biol..

